# Oral Vaccination with *Aeromonas veronii* Ghost Against Lethal Bacterial Infection in *Cyprinus carpio*

**DOI:** 10.3390/vaccines13090985

**Published:** 2025-09-20

**Authors:** Na Jiang, Zhihong Ma, Jinjing Zhang, Lin Luo, Xingchen Huo, Jufeng Jiang, Jian Gao, Jie Huang, Dongjie Shi

**Affiliations:** 1 Fisheries Science Institute, Beijing Academy of Agriculture and Forestry Sciences, Beijing 100068, China; jiangna@baafs.net.cn (N.J.); mazh@baafs.net.cn (Z.M.); zhangjinjing@baafs.net.cn (J.Z.); luolin@baafs.net.cn (L.L.); huoxingchen@baafs.net.cn (X.H.); 2 Key Laboratory of Urban Agriculture (North China), Ministry of Agriculture and Rural Affairs, Beijing 100097, China; 3 National Engineering Research Center for Freshwater Fisheries (Beijing), Beijing 100068, China; 4 Tianjin Fisheries Research Institute, Tianjin Ornamental Fish Technology and Engineering Centre, Tianjin 300221, China; jufengjiang@163.com; 5 College of Fisheries, Huazhong Agricultural University, Wuhan 430070, China; gaojian@mail.hzau.edu.cn

**Keywords:** *Aeromonas veronii*, bacterial ghost, oral vaccination, innate immune, acquired immune

## Abstract

Background/Objectives: *Aeromonas veronii* is a significant pathogen affecting aquatic animals and has the potential to infect humans. Vaccination remains the most effective strategy for preventing infections caused by this bacterial strain. Methods: This study aimed to validate the efficacy of bacterial ghosts as an oral vaccine by administering them to *Cyprinus carpio* and evaluating the resultant innate and acquired immune responses. Following immunization, the vaccinated *Cyprinus carpio* were exposed to a lethal dose of the wild-type bacterial strain to assess survival rates and relative protection efficiency. Results: Oral vaccination with bacterial ghosts led to the significant enhancement of lysozyme (LZM) and myeloperoxidase (MPO) activity in koi serum. It also resulted in the upregulation of cytokines, such as IL-2 and TNF-α, as well as an increase in both systemic (IgM) and mucosal (IgZs) antibody responses. The immunized group demonstrated reduced cumulative mortality following bacterial challenge. The relative percent survival in the vaccinated group reached as high as 87.50%. Conclusions: The oral immunization of *Cyprinus carpio* with *A. veronii*-derived bacterial ghosts confers substantial immune protection, providing a foundational basis for the development of novel vaccines against *A. veronii*.

## 1. Introduction

*Aeromonas veronii* is a Gram-negative, facultatively anaerobic short bacillus that is extensively distributed in freshwater, soil, and the bodies of aquatic animals [1]. As a significant member of the *Aeromonas* genus, *A. veronii* has garnered sustained attention due to its multifaceted roles in zoonotic diseases, aquaculture diseases, and environmental microbial ecology [2]. In particular, within the aquaculture sector, this bacterium is a primary pathogen responsible for causing hemorrhagic septicemia in freshwater fish, such as crucian carp and common carp, resulting in substantial economic losses globally [3]. Recent studies have also identified that *A. veronii* harbors multiple drug-resistant genes, and its resistance to β-lactam and tetracycline antibiotics has exacerbated the challenges associated with its prevention and control [4].

In recent years, the detailed investigation of the pathogenic mechanisms and virulence factors of *A. veronii* has underscored the significance of vaccine development as a pivotal strategy for managing infections caused by this pathogen. Current research efforts in aquaculture vaccines targeting *A. veronii* have yielded notable advancements, encompassing a range of vaccine types, including inactivated, attenuated, subunit, and nanovaccines [5,6,7,8,9]. Nonetheless, despite these advancements, numerous challenges persist. Primarily, the complexity and diversity of *A. veronii* virulence factors pose a significant obstacle, as vaccines based on a single antigenic component may fail to confer comprehensive protection. Therefore, attenuated and inactivated vaccines remain the key focus operations; oral vaccines have always been an ideal formulation for fish vaccines. Therefore, another area of focus for researchers is the development of oral vaccines. Chen et al. utilized *Lactobacillus casei* to express the antigen Aha1 fused with CTB, constructing a probiotic-based oral vaccine. Oral vaccination with this vaccine can protect *common carp* (*Cyprinus carpio*) from infection with *A. veronii* [5]. A study from the Philippines shows that oral vaccination with the inactivated *A. veronii* DFR01 (Diseased Fish Rizal) can elicit improved immune responses in fish and result in higher survival rates in the motile aeromonad septicemia model [10].

Bacterial ghost vaccine is an innovative form of vaccine. It is produced by removing components such as nucleic acids from bacteria through specific methods, while retaining the intact cell wall structure of the bacteria [11]. This type of vaccine has unique advantages. On one hand, bacterial ghosts preserve the surface antigens of bacteria, which can effectively stimulate the body’s immune system to produce an immune response. On the other hand, the removal of nucleic acids eliminates the pathogenicity of bacteria, making it relatively safe. It shows great potential in preventing bacterial infectious diseases and can be researched and developed for various pathogenic bacteria, providing a new approach and strategy for the prevention and control of infectious diseases [12,13,14].

In our previous work, using the *A. veronii* isolate from naturally ulcer koi, we constructed a bacterial ghost vaccine, and this vaccine could stimulate both innate immune and specific immune responses in *Cyprinus carpio*. At the same time, this bacterial ghost vaccine provided strong immune protection for koi, especially those infected by *A. veronii* [15]. Nevertheless, whether oral immunization with this vaccine can retain satisfactory immunogenicity and protective effectiveness remains a compelling issue worthy of in-depth investigation.

In this work, using *Cyprinus carpio* koi as an animal model, we evaluated the oral immunization efficacy of the previously constructed *A. veronii* ghost vaccine. Our results provide some useful experimental evidence for the development of an oral *A. veronii* ghost vaccine and potential strategies for the aquaculture industry to combat *A. veronii* infections.

## 2. Materials and Methods

### 2.1. Preparation of A. veronii Ghost (AVG) Vaccine

The *A. veronii* ghosts were prepared according to previous studies [15]. Briefly, A single colony of the engineered *A. veronii* (harboring plasmid pBBR1MCS2-E) was picked and incubated in 10 mL LB containing 50 μg/mL kanamycin, followed by overnight incubation at 28 °C. After overnight incubation, subculture the engineered bacteria by transferring 1 mL of culture into 100 mL of fresh LB broth (50 μg/mL kanamycin) in a sterile 250 mL flask. Incubate at 28 °C with shaking at 220 rpm until mid-log phase (OD_600_ ≈ 0.4–0.6). Then, the cultivation temperature was raised to 42 °C to induce bacterial ghost formation. Approximately 16 h later, centrifuge to collect the bacterial ghosts, and verify that the qualified lysis rate meets standards for subsequent experimental fish immunization. Specifically, the lysis rate was carried out following the method described in our previous study [15]. The plate counting method was used for detection, and the results confirmed that the lysis rate of bacterial ghosts reached the 100% standard, with no viable bacterial cells detected.

### 2.2. Feed

The koi feed used in this trial was purchased from Beijing Friendship Hengyuan Technology Co., Ltd., Beijing, China. The control group was fed with standard koi feed. For the high-dose bacterial ghost group, the bacterial ghost suspension was added to the feed to achieve a final concentration of 5.9 × 10^7^ cells per gram of feed (cells/g); for the low-dose group, the final concentration of AVG in the feed was set at 2.9 × 10^7^ cells/g. The specific preparation process was as follows: First, the AVG suspensions were prepared in phosphate-buffered saline (PBS) to obtain the different final concentrations. Second, the suspension was uniformly sprayed onto koi feed, which was then thoroughly mixed using a small-scale mixer. After mixing, the feed was air-dried in shade.

### 2.3. Cyprinus carpio Immunization

This study has been reviewed and approved by the Institutional Ethics Committee of Beijing Academy of Agriculture and Forestry Sciences. A total of 270 healthy *Cyprinus carpio* (35 ± 0.2 g) were obtained from an aquaculture farm in Beijing, China. Fish were acclimated in plastic tanks (30 fish in each tank) with water temperature maintained at 24.0 ± 1.0 °C. Three experimental groups were established: one control group, one low-dose AVG group, and one high-dose AVG group. Each group was set up with 3 replicate tanks. The immunization was administered using the mixed-feed method. After the trial commenced, the AVG groups received the experimental feed for two weeks (2 wk) per immunization cycle, with two-week intervals followed by another immunization cycle. The control group was continuously fed the control diet throughout.

Samples were collected on days 7, 14, 21, 28, 35, 42, and 49. For each group, three fish were randomly selected, and blood was drawn from the caudal vein after being anesthetized with MS-222. A 100 μL aliquot of whole blood was mixed with sodium heparin anticoagulant for the respiratory burst assay. The remaining blood samples were centrifuged at 1000× *g* for 10 min at 4 °C to collect serum, which was then stored at −80 °C for subsequent non-specific immune parameter and serum titer analyses. To obtain the intestinal mucosal sample for the antibody test, 200 μL of intestinal mucus was sampled from each fish, and an equal volume of phosphate-buffered saline (PBS) was added to the mucus sample. Intestinal mucus samples were then transferred into centrifuge tubes for storage at −80 °C.

### 2.4. Innate Immune Response Assessment

#### 2.4.1. Determination of Enzyme Activity in Serum

Myeloperoxidase (MPO) activity in serum was measured using commercial test kits (Nanjing Jiancheng Bioengineering Institute, Nanjing, China) according to the manufacturer’s protocols. The lysozyme (LZM) activity was measured using a turbidimetric assay [15]. One unit of lysozyme activity was defined as the amount of sample causing a reduction in absorbance of 0.001/min. All measurements were performed at least in triplicate.

#### 2.4.2. Detection of Cellular Immune-Related Factors

Commercially available ELISA kits (Cusabio, Wuhan, China) were utilized to quantitatively detect the levels of IL-2, IL-4, IL-10, and TNF-α cytokines in serum samples from immunized animals according to the manufacturer’s instructions, with three technical replicates performed for each sample.

### 2.5. Antibody Titers Assessment

#### 2.5.1. Agglutination Test for Serum Antibody Titers

A 96-well plate hemagglutination test was performed using a two-fold serial dilution method for serum. For each well, 50 μL of serum was added, followed by the addition of 50 μL of CL0901 bacterial suspension (2 × 10^7^ CFU/mL). The wells with only the bacterial suspension were used as a control. The agglutination reaction was carried out at room temperature and observed after 24 h. Results were judged by visual inspection and microscopy; an uneven edge clump at the U-shaped bottom of the well indicated a positive result, while a clear, round precipitate indicated a negative result. The highest dilution showing a clear positive reaction was recorded as the serum titer.

#### 2.5.2. ELISA for Measuring Serum IgM Antibody Titers

The antigen was diluted to a concentration of 5 μg/mL in coating buffer and added to the reaction plate (100 μL per well) to coat the 96-well plate overnight at 4 °C. Each well was then washed three times with 300 μL of PBST wash buffer for 5 min each time. Blocking was done by adding 200 μL of PBST containing 1% BSA to each well and incubating at 37 °C for 2 h. After washing with PBST three more times, the carp serum samples were serially diluted in diluent buffer and added to the reaction plate (100 μL per well, three wells per dilution). Negative controls included primary antibodies with control carp serum, and blank controls included primary antibodies with diluent buffer alone. Plates were incubated at 37 °C for 2 h. Following another three washes with PBST, a rabbit anti-carp serum IgM secondary antibody diluted 4000-fold in diluent buffer was added (100 μL per well) and incubated at 37 °C for 1 h. TMB substrate solution (100 μL per well) was added and allowed to develop color in the dark at room temperature for 10–20 min until blue. The reaction was stopped by adding the stop solution (100 μL per well), and the plates were left to stand for 5 min, turning the solution from blue to yellow. OD values were measured at 450 nm using a microplate reader. The average OD450 value of unimmunized carp serum served as the negative control value. A P/N ratio ≥ 2.1 was considered positive. The antibody titer was determined based on the dilution factor of the carp serum.

#### 2.5.3. ELISA for Measuring Intestinal Mucus IgZ1, IgZ2, and IgZ3 Antibody Titers

The procedure is essentially the same as described in Section 2.5.2, but instead of serum samples, intestinal mucus samples were used. Secondary antibodies specific for carp IgZ1, IgZ2, and IgZ3 (produced by AbMax Biotechnology Co., Ltd., Beijing, China) were employed for detection.

### 2.6. Immune Protection Rate Determination Experiment

A total of 96 *Cyprinus carpio* were divided into 12 groups, with 6 groups serving as controls and the other 6 groups immunized with a 10% mass ratio of AVG suspension (concentration: 5.9 × 10^8^ cells/mL). Four weeks after completing the second cycle of bacterial inclusion feeding, an infection experiment was conducted on the experimental fish to evaluate the immune protection rate. The challenge was performed using *A. veronii* strain CL0901 at two different concentrations: a high concentration of 9.3 × 10^6^ CFU/mL (about 2LD_50_) and a low concentration of 9.3 × 10^4^ CFU/mL. Each fish received an intraperitoneal injection of 0.2 mL of the bacterial suspension. Three immunized groups and three control groups were challenged with low-dose bacteria, while the remaining three immunized groups and three control groups were challenged with the high-dose bacteria. The fish were continuously observed for 14 days, and mortality was recorded. The relative percent survival (RPS) was calculated using the formula: RPS = (1 − Mortality rate in immunized group/Mortality rate in control group) × 100%.

### 2.7. Statistical Analysis

All data were represented as mean ± standard deviation (SD) and analyzed using GraphPad Prism 9.5.0.

To compare the differences in enzyme activity, cytokines, and specific antibodies in serum among groups at the same time point, the normality of data was first assessed using the Shapiro–Wilk test to verify whether the data conformed to a normal distribution. For data that met the normal distribution (Shapiro–Wilk test, *p* > 0.05), One-way analysis of variance (ANOVA) was performed to test for overall differences among groups, followed by Tukey’s Honestly Significant Difference (HSD) test for pairwise comparisons between three groups. For non-normally distributed data (Shapiro–Wilk test, *p* ≤ 0.05), the Kruskal–Wallis test was used instead to compare differences among groups. statistical significance was denoted by asterisks: * *p* < 0.05, ** *p* < 0.01, and *** *p* < 0.001.

For the analysis of IgZs titiers and fish motality rate, the same Shapiro–Wilk test was first applied to verify data normality. Subsequent comparative analysis was performed using the two-tailed Student’s *t*-test, with statistical significance indicated by asterisks: * *p* < 0.05 and *** *p* < 0.001.

## 3. Results

### 3.1. Enzyme Activity in Serum

**Figure 1:**
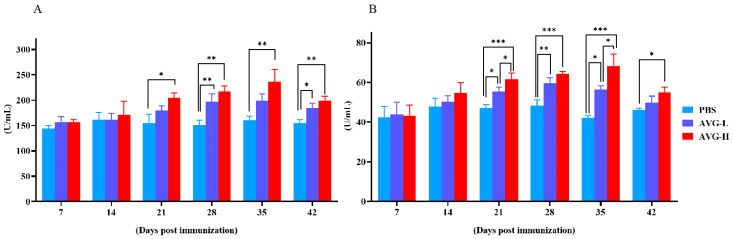
Lysozyme and myeloperoxidase activity. (**A**). Lysozyme activity in immunized serum. (**B**). Myeloperoxidase activity in immunized serum. AVG-L, low-dose group; AVG-H, high-dose group. Values are expressed as mean of each group (sample size, *n* = 3) ± SD. As data met the normal distribution (Shapiro–Wilk test, *p* > 0.05), statistical comparisons among the various treatment groups at each time point were performed utilizing Tukey’s HSD test. * *p* < 0.05, ** *p* < 0.01, *** *p* < 0.001.

By Day 21, the serum lysozyme (LZM) levels in the high-concentration AVG-immunized group were significantly elevated compared to the control group, while the low-concentration AVG-immunized group did not exhibit a statistically significant difference in LZM levels relative to the controls (Figure 1A). On Days 28 to 42, both high- and low-concentration AVG-immunized groups demonstrated significantly higher myeloperoxidase (MPO) levels compared to the control group. Furthermore, from Day 21 onward, there was a significant increase in MPO levels in both AVG-immunized groups relative to the control group (Figure 1B).

### 3.2. Cytokines in Serum

**Figure 2:**
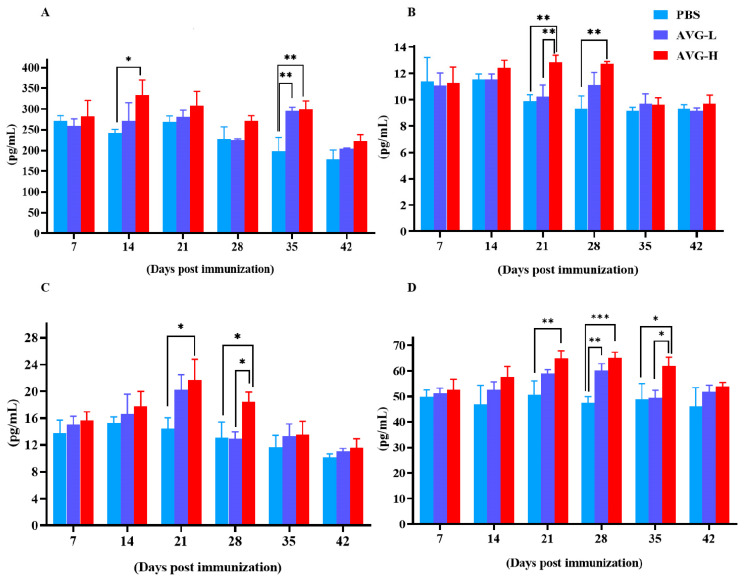
Cytokine levels in Cyprinus carpio serum after immunization with AVG. (**A**). IL-2; (**B**). IL-4; (**C**). IL-10; (**D**). TNF-α. AVG-L, low-dose group; AVG-H, high-dose group. Values are expressed as mean of each group (sample size, *n* = 3) ± SD. As data met the normal distribution (Shapiro–Wilk test, *p* > 0.05), statistical comparisons among the various treatment groups at each time point were performed utilizing Tukey’s HSD test. * *p* < 0.05, ** *p* < 0.01, *** *p* < 0.001.

Simultaneously, serum levels of cytokines, including IL-2, IL-4, IL-10, and TNF-*α* were measured using ELISA kits. The results demonstrated significantly elevated cytokine concentrations in the AVG-immunized koi group compared to the control group. The peak concentration of cytokines generally occurred between 21 and 28 days post-immunization except for IL-2 levels in the high-dose group, which exhibited a significant elevation on day 14 post-immunization (Figure 2). However, significant differences in cytokine levels between the low-dose and high-dose groups were only observed at specific time points, including IL-4 on day 21 (Figure 2B), IL-10 on day 28 (Figure 2C), and TNF-α on day 35 (Figure 2D).

### 3.3. Serum Antibody

**Figure 3:**
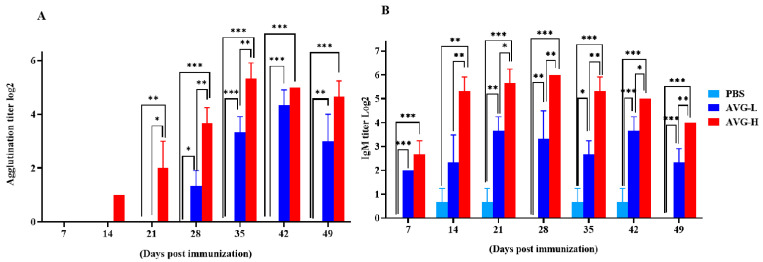
Serum antibody titers in koi vaccinated with AVG. (**A**). Agglutination titers; (**B**). IgM titers. AVG-L, low-dose group; AVG-H, high-dose group. Values are expressed as mean of each group (sample size, *n* = 3) ± SD. As data met the normal distribution (Shapiro–Wilk test, *p* > 0.05), statistical comparisons among the various treatment groups at each time point were performed utilizing Tukey’s HSD test. * *p* < 0.05, ** *p* < 0.01, *** *p* < 0.001.

The serum agglutination titers in *Cyprinus carpio* that were orally immunized with ghost bacteria were significantly elevated compared to those in the control group on Days 14, 28, 35, 42, and 49. The peak serum agglutination and IgM titers reached values of 2^4.33^ and 2^5.33^ in the low- and high-dose groups, respectively. Similarly, serum IgM titers were significantly higher in the immunized group than in the controls on Days 7, 14, 21, 35, 42, and 49. Moreover, the IgM levels in the high-dose group were significantly higher than those in the low-dose group at most time points post-immunization (Figure 3). These findings suggest that oral immunization with ghost bacteria induces a sustained enhancement of systemic antibody responses in *Cyprinus carpio*, with antibody titers remaining at elevated levels.

### 3.4. Mucosal Antibody Levels

**Figure 4:**
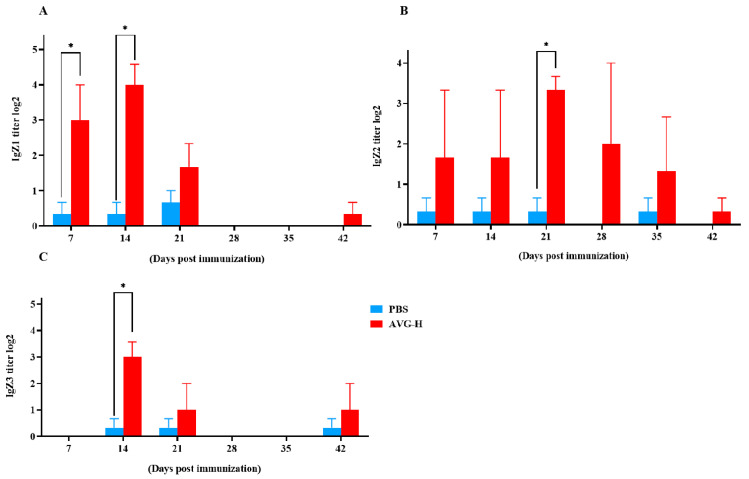
Mucosal antibody levels in the intestinal mucus of koi vaccinated with AVG. (**A**). IgZ1 titers; (**B**). Ig Z2 titers; (**C**). Ig Z3 titers.AVG-H, high-dose group. Values are expressed as mean of each group (sample size, *n* = 3) ± SD. As data met the normal distribution (Shapiro–Wilk test, *p* > 0.05), statistical comparisons between the two groups at each time point were performed utilizing two-tailed Student’s *t*-test. * *p* < 0.05.

Mucosal antibody levels in the intestinal mucus of orally immunized koi were enhanced (As presented in Figure 4). Intestinal mucosal IgZ1 antibody titers were significantly higher in the immunized group compared to controls on Days 7 and 14. Mucosal IgZ2 antibodies exhibited significant elevation in the immunized group on Day 21, while mucosal IgZ3 antibodies were significantly increased on Day 14. The peak titers were 2^4.00^ for IgZ1, 2^3.33^ for IgZ2, and 2^3.00^ for IgZ3. These data indicate that during the early stages of immunization, ghost bacteria stimulate the production of mucosal antibodies. Thus, during the early stages of oral vaccination with ghost bacteria, the vaccine stimulated antibody production in the intestinal mucosa of *Cyprinus carpio*. This response encompassed all three known subtypes of IgZ antibodies, with mucosal antibody titers ranging from 2^3^ to 2^4^.

### 3.5. Cumulative Mortality

**Figure 5:**
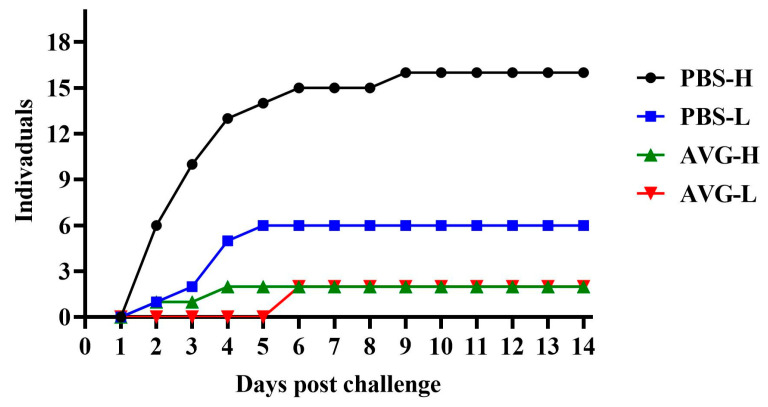
Mortality dynamics following wild-type pathogen challenge in immunized subjects. PBS-H and PBS-L, PBS control groups challenged with a high or low dose of bacteria; AVG-H and AVG-L, vaccinated groups challenged with a high or low dose of bacteria.

The cumulative mortality data for the experimental animals are presented in Figure 5. When challenged with a high concentration of *A. veronii*, the AVG-immunized group experienced only two mortalities during the initial four days of the challenge. Conversely, the control group exhibited a significantly higher mortality rate, with 13 deaths occurring within the same early challenge period, and continued to experience fatalities during the mid-challenge phase. These findings suggest that AVG confers protection against high-concentration virulent strains. In the context of a low-concentration *A. veronii* challenge, the AVG-immunized group did not experience any mortalities until the seventh day, at which point two deaths were recorded. In contrast, the control group had already accumulated six mortalities by the fifth day of the challenge. These results indicate that, under a low-concentration challenge, AVG not only delayed the onset of mortality in *Cyprinus carpio* but also reduced the overall number of deaths.

### 3.6. Relative Percent Survival (RPS)

**Figure 6:**
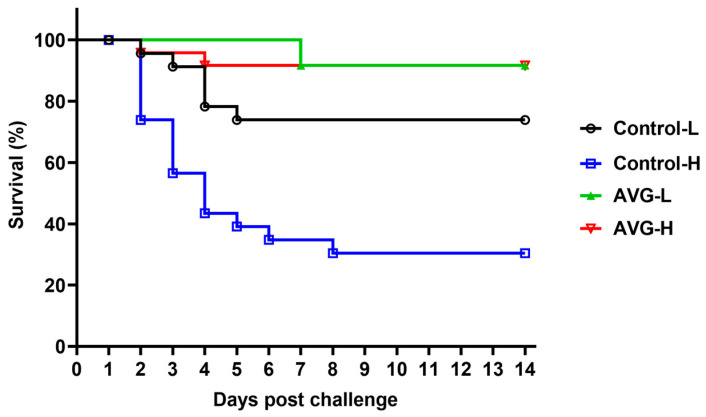
Survival curve analysis following *A. veronii* challenge. PBS-H and PBS-L, the PBS control groups challenged with a high or low dose of bacteria; AVG-H and AVG-L, the vaccinated groups challenged with a high or low dose of bacteria.

As illustrated in Table 1, the AVG-immunized cohort demonstrated a significantly enhanced survival rate compared to the PBS control group, regardless of the *A. veronii* challenge concentration. Notably, within the high-concentration *A. veronii* challenge cohort, the control group exhibited a substantially elevated average mortality rate of 66.67% ± 4.17%, while the AVG-immunized group recorded an average mortality rate of merely 8.33% ± 4.17%, corresponding to a relative percent survival rate of 87.50%. Similarly, in the low-concentration *A. veronii* challenge cohort, the AVG-immunized group achieved an average mortality rate of 8.33% ± 4.17%, resulting in a relative percent survival rate of 66.68%. The summarized survival rates across all dose groups are presented in Figure 6.

## 4. Discussion

In this study, we conducted a preliminary evaluation of the oral immunization efficacy of the *A. veronii* ghost vaccine. Our results demonstrate that this vaccine not only induced the expression of multiple innate immune-related factors but also stimulated the antigen-specific humoral responses, including both systemic IgM and mucosal IgZs. These findings suggest that oral immunization with the AVG vaccine may represent an effective strategy for preventing *A. veronii* infection in *Cyprinus carpio*.

In mammals, the principal routine function of IL-10 is to limit and ultimately terminate inflammatory responses [16]. IL-10 is an anti-inflammatory cytokine that plays a critical role in the control of immune responses [17]. In fish, IL-10 may also be correlated with anti-inflammatory properties and may play a significant role in fish disease [18]. In this study, we found that IL-10 in the serum of the treated koi was highly induced. Similar results were observed in zebrafish orally administered with a nanoparticle loaded outer membrane protein A (ompA) from *A. hydrophila* and in tilapia orally vaccinated with *A. hydrophila* omp23-based nanoparticle [19,20]. As IL-10 mRNA transcripts could be up-regulated during aeromoniasis following intraperitoneal injection of *A. hydrophila* in Indian major carp (*Catla catla*) [18], we hypothesized that the induction of IL-10 expression in the present study would follow the same pattern. In the mammalian immune system, IL-10 was the only cytokine capable of inducing large amounts of IgA, as well as some IgG, by simulating naive B cells in the presence of anti-CD40 Ab [21]. Therefore, we hypothesize that higher IL-10 concentrations correlate with elevated titers of IgM and IgT/D, which represent systemic and mucosal immunity in fish, respectively.

Fish TNFs participate in regulating immune function, inflammation, metabolism, and morphological development. Some studies demonstrated up-regulated expression of TNF-α in vivo after stimulation with different bacterial infections in the early inflammation stage [22,23,24]. Our bacterial ghosts were orally administered daily to koi for two weeks as a vaccine, and we found that TNF-α production was induced at 21st day post-vaccination (dpv) and persisted for 2 weeks. In this study, the continuous stimulation with *A. veronii* ghosts elicited the marked elevation of TNF-α, albeit with a delayed onset compared to the acute inflammatory response induced by a single exposure to live bacteria or LPS. This observation was nearly consistent with the findings of two separate studies: Mave Harshitha et al. detected an upregulation of TNF-α in zebrafish (*Danio rerio*) on the 50th dpv, while Abhiram et al. observed an elevation in TNF-α expression in tilapia from the 7th dpv to the 28th dpv [20,25]. Specifically, the zebrafish in their study received consecutive 51-day oral administration of *A. hydrophila* outer membrane protein A (OmpA)-loaded nanoparticles, while the tilapia were given two rounds of 3 consecutive-day oral administration of *A. hydrophila* outer membrane protein (OMP)-loaded nanoparticles, respectively. TNF-α exerted an impact on fish mucosal immunity by upregulating the IgT gene at the hindgut of sea bass vaccinated and adjuvanted with rTNF-α [26]. A similar stimulatory effect on mucosal immunity may also occur in koi vaccinated with *A. veronii* ghosts.

Beyond innate immunity above, there is another critical component of the immune system: adaptive immunity, which encompasses both humoral and mucosal immunity. The IgM titer is one of the most important parameters of humoral immunity. Koi vaccinated with AVG have a higher IgM titer from 7th dpv to 49th dpv than the control group. Many studies have detected similar changes in either the transcriptional levels of IgM expression or in the IgM titers measured by ELISA. Tilapia orally immunized with a nanoparticle-based vaccine containing inactivated whole-cell bacteria exhibited upregulated IgM gene expression in the spleen, kidney, and gut [25]. Consistent findings have also been observed in ELISA-based detections. In the research of African catfish [27], following oral vaccination with *A. veronii*, the fish showed significantly higher IgM titers from 2 weeks post-vaccination (wpv) to 4 wpv, with the peak titer reaching 2^4.42^, which is very close to the 2^5.33^ observed in the present study. In another research, where a whole-cell *A. hydrophila* biofilm vaccine was evaluated in rohu, the orally vaccinated group showed increased antibody titer from 0th dpv to 50th dpv [28]. Furthermore, the antibody isotype IgT/IgZ represents the most critical parameter for assessing mucosal immunity in many teleost fish and is reported to play a role in gut-associated immune responses [29,30,31]. Notably, common carp (*Cyprinus carpio*) expresses three IgZ subclasses: IgZ1 is more abundant in systemic organs, IgZ2 is preferentially expressed at mucosal sites [32], and IgZ3 expression is strongly induced in the hindgut during *A. hydrophila* challenge by immersion [33]. To date, there have been few reports evaluating the effects of oral vaccination on fish mucosal immunity by detecting all three IgZ subclasses via ELISA. In the present study, we detected elevated titers of these three IgZ subclasses at different time points post-vaccination, with the upregulation persisting for one to two weeks. Consistent findings have been reported in another study: following administration of encapsulating chitosan-loaded DNA vaccine, an increase in IgT levels was observed in the spleen and hindgut of fish [34].

This study focuses on the changes in innate and adaptive immunity of koi induced by AVG, with the detection of the enzyme activity, cytokine activity, and antibody titers. However, the specific mechanism by which AVG stimulates the production of IgM and IgZ antibodies remains unclear. To fill this research gap, the subsequent studies will utilize transcriptomic analysis, immunohistochemistry, and Western blotting to further investigate the pathways through which the vaccine enhances immune protection in fish against bacterial challenges. Additionally, further research is required to determine the optimal dosing regimen and balance the vaccine immunogenicity with biosafety considerations.

## 5. Conclusions

In conclusion, this study provides preliminary evidence that the bacterial ghost vaccine derived from *A. veronii* possesses both innate and acquired immune responses. Our study provides an experimental foundation for the development of novel oral fish vaccines against *A. veronii*.

## Figures and Tables

**Table 1:** Protective immunity induced by the AVG vaccine in *Cyprinus carpio* challenged with graded doses of *A. veronii*.

Challenge Concentration (CFU/mL)	Group	Number	Deaths	Mortality Rate (%)	Mean Mortality Rate (%)	RPS (%)
9.3 × 10^6^	AVG	8	1	12.5	8.33 ± 4.17 ***	87.5
		8	1	12.5		
		8	0	0		
	PBS	8	6	75	66.67 ± 4.17	/
		8	5	62.5		
		8	5	62.5		
9.3 × 10^4^	AVG	8	1	12.5	8.33 ± 4.17 *	66.68
		8	0	0		
		8	1	12.5		
	PBS	8	2	25	25.00	/
		8	2	25		
		8	2	25		

Note: Values are expressed as mean of each group (sample size, *n* = 3) ± SD. As data met the normal distribution (Shapiro–Wilk test, *p* > 0.05), statistical analysis between the two groups with the same challenge dose was performed using two-tailed Student’s *t*-test. * *p* < 0.05 and *** *p* < 0.001.
